# Acclimatising with 3D virtual reality video improves simulator performance: initial findings of a randomised controlled feasibility trial

**DOI:** 10.1007/s00464-025-12153-x

**Published:** 2025-09-10

**Authors:** T. Shakir, G. Lingam, N. Francis, M. Chand

**Affiliations:** 1 Department of Surgery & Interventional Science, University College London, Gower St, London, WC1E 6BT UK; 2 Colorectal Surgery, St Mark’s Hospital and Academic Institute, London, UK; 3 The Griffin Institute, London, UK

**Keywords:** Virtual reality, Three dimensional, Video, Robotic, Simulator, Performance

## Abstract

**Introduction:**

The transition from traditional laparoscopy to robotic surgery marks a significant chage in surgical practice. An understated aspect of this transition may be the three dimensional (3D) view from the surgical console. This study hypothesises that acclimatisation with 3D virtual reality (VR) video may enhance robotic simulator performance in novice robotic surgeons.

**Methods:**

This feasibility randomised controlled trial (RCT) involved 18 participants, randomly assigned to either a 3D VR video group or a 2D video group. The 3D group viewed a procedural video on a VR headset, while the 2D group watched the same video on a standard laptop screen. Participants then performed the initial 4 introductory robotic simulator exercises. Primary outcomes included automated performance metrics (APMs) including instrument path length, completion time, penalty scores, and overall performance score. Secondary outcomes were perceived mental workload using the NASA Task Load Index (NASA TLX) and cybersickness rates.

**Results:**

The 3D VR group demonstrated significantly better performance across all primary outcome measures. Mean overall performance scores for 3D VR was 52.75, compared to 29.78 for 2D (p < 0.01) Mean instrument path length for the 3D VR group was 305.09 cm, compared to 413.72 cm for the 2D group (p < 0.01) The 3D VR group incurred fewer penalty scores, with a mean of -8.16 compared to—23.99 for the 2D group (p = 0.03). 3 participants (21.4%) reported mild cybersickness symptoms with VR, which were transient. No significant differences were observed in perceived mental workload between the groups.

**Conclusion:**

Acclimatisation with 3D VR video significantly enhances simulator performance among novice robotic surgeons, suggesting its potential integration into standard robotic surgery training protocols. Further studies with larger sample sizes and clinical settings are warranted to confirm these findings.

The transition from traditional laparoscopy to robotic surgery represents a significant stride in surgical practice. This is primarily due to the additional controls and features offered by a robotic platform. Bespoke training courses over multiple days allow one to become immersed in all these aspects [1]. Whilst online learning can aid a hybrid approach to training, and possibly flatten learning curves [2], there are features which cannot routinely be acquired in advance. One often underestimated aspect is the three dimensional (3D) view from the surgical console. With a lack of haptic feedback, it is postulated that more onus is placed on the view, with visual cues compensating for a lack of tactile perception [3]. All robotic surgical systems utilise an endoscope with two lenses which provides a left and right video stream. Combining these together affords the user a 3D view—either from a closed console, or an open console whilst wearing 3D glasses.

However, adapting to 3D surgery is not without its challenges, particularly for novices who must adjust to the three-dimensional (3D) views provided, in contrast to the two-dimensional (2D) laparoscopic screens they are accustomed to. The ability to effectively interpret and navigate these 3D views is crucial for optimising surgical performance and patient outcomes. Given the ubiquity of 2D screens in both professional and everyday contexts, there may be a natural learning curve associated with acclimatising to 3D digital environments. Overcoming this learning curve could be an additional factor in increasing efficiency.

The initial phase of robotic training invariably involves simulation. This allows a safe environment to become accustomed to using the controls. There is a requirement by many robotic platforms to achieve certain simulator score thresholds, with learning curves described as ranging up to 80 hours [4, 5]. To address this, our study hypothesised that a period of acclimatisation with 3D virtual reality (VR) video could enhance simulator performance in novices. Our unit has developed an innovative approach to this challenge by extracting stereoscopic video from the robotic console and integrating it into a VR headset. This setup provides a 3D perspective akin to that experienced from within a closed console. Novices and trainees can therefore familiarise themselves with the 3D visual environment in a controlled, simulated setting. By doing so, we aim to reduce the initial cognitive load and improve overall performance during the early stages of robotic surgery training.

## Methods

### Trial design

This study was designed as a feasibility randomised controlled trial (RCT) to evaluate the impact of 3D virtual reality (VR) video on simulator performance among novice robotic surgeons. The design was chosen owing to the fact that this new technology has not yet been evaluated for this purpose. We anticipate there may be certain issues surrounding the implementation, with novel technology, potential for cybersickness and hardware constraints possible. As such, a feasibility study was conducted to ensure that the intervention is viable and the study design robust. This aids with resource allocation and organisation, forming the foundation for a subsequent trial.

The study was conducted on a clinically used da Vinci Xi system. Appropriate approval was received from operating room management to use the equipment out of hours (e.g. Sundays) when then system was not being used. The study coordinator had received appropriate training in managing the robotic equipment. The CONSORT (Consolidated Standards of Reporting Trials) statement was followed [6] and ethical approval was received from the institutional review board (Project ID: 26,587/001).

### Participants

Inclusion and exclusion criteria are listed in Table 1.  Participants were recruited through word of mouth—social media adverts and posters. Written, informed consent was obtained from all participants prior to their involvement in the study.

**Table 1:** Inclusion and exclusion criteria

Inclusion criteria	Exclusion criteria
National Health Service (NHS) staff—Medical students to consultant/attending surgeons	Prior experience with robotic surgical console
Robotic surgery novices	

### Intervention

After informed consent, participants were assigned to one of two groups: 3D or 2D video group. The 3D video group watched a procedural video (robotic anterior resection) on a VR headset for 20 min. The 2D video group watched the same procedural video in 2D, but on a standard laptop screen.

Following 2D or 3D video, participants evaluated their perceived mental workload using the NASA Task Load Index (NASA TLX) and evaluated their cybersickness. This was repeated at the end of the simulator session. A short introduction to the robotic console controls was given of less than 5 min duration. Participants then performed a structured series of simulator exercises on the Intuitive da Vinci Xi SimNow platform, which were the initial four introductory tasks (Table 2).

**Table 2:** Simulator tasks

Task name	Description
	A colour coordinated pick and place task
	Passing rings along a tortuous wire without touching
	Utilising the degrees of freedom of the hand controllers to touch targets
	Manoeuvring the robotic camera to centre on targets in 3D space around a virtual environment

### Outcomes

The primary outcome measure was the automated performance metrics (APM’s) obtained from the simulator. These metrics are displayed in Table 3.

**Table 3:** Simulator automated performance metrics

Metric	Description
Instrument path length	The total distance travelled by the robotic instruments during the tasks in centimetres (cm)
Completion time	The total time taken to complete all tasks in seconds
Penalty scores	The number of errors or deviations from the optimal path within each task-specific requisite
Overall performance score	A composite score reflecting overall performance, based on the above

The secondary outcomes were perceived mental workload, assessed using the NASA Task Load Index (NASA TLX) [7], in addition to qualitative analysis of cybersickness. Participants completed the NASA TLX questionnaire at two time points—after watching the video and after completing the simulator tasks.

### Sample size

Owing to the study design of a feasibility RCT, a sample size calculation is not applicable. The primary purpose of the trial is to ascertain factors pertaining to the novel technology and software, including logistics and hardware constraints. A pragmatic approach was therefore adopted with regards to a sample size of 18 participants to test the feasibility as well as to provide a power calculation for further larger study. This allows for 3 sessions utilising the robotic theatre when the system is not in clinical use.

### Randomisation

After appropriate informed consent, participants were randomly allocated into one of two groups—3D VR vs 2D. An online tool was used to create a block randomisation sequence with a block size of 6, and an allocation ratio of 1:1 [8].

### Data analysis

Descriptive statistics were used to summarise participant demographics and baseline characteristics, in addition to cybersickness rates. Independent t-tests were conducted to compare the mean scores between the two video groups, and NASA TLX scores. A p-value of less than 0.05 was considered statistically significant.

## Results

### Participant flow

**Fig. 1:**
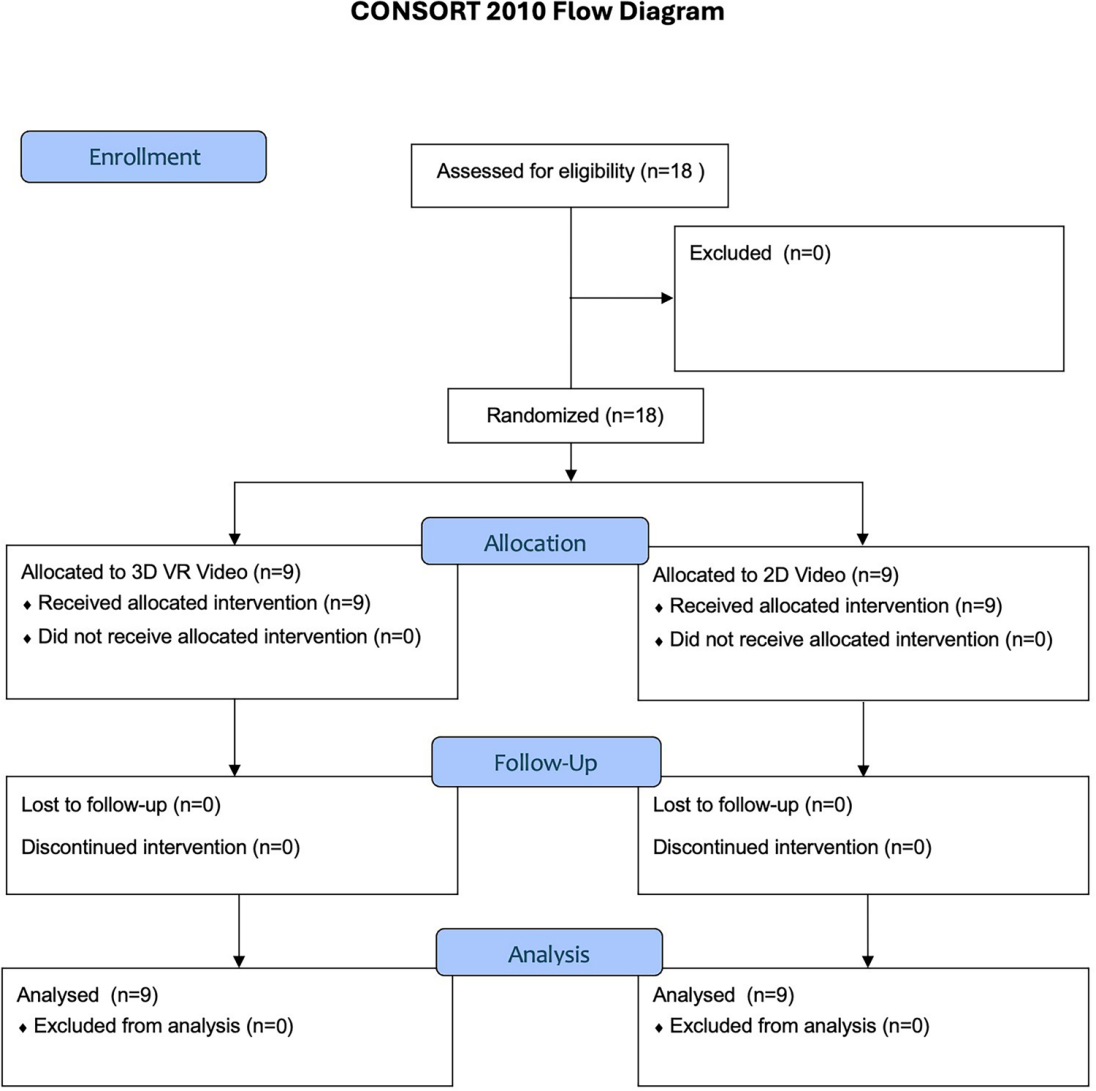
CONSORT flow diagram

Figure 1 describes the flow with a total of 18 participants enrolled in the study. 9 participants were assigned to the 3D VR video group and 9 to the 2D video group.

### Baseline data

The demographic characteristics of the participants were comparable between the two groups, with no significant differences in age, gender, or prior surgical experience (Table 4). The mean age of participants was 28.5 years (range: 24–43 years), and the cohort included 9 males and 9 females.

**Table 4:** Participant demographics

		**3D**	**2D**	
N		9	9	
Age		29.6	30.5	p = 0.70
Gender	M	4	5	
	F	5	4	
Handedness	R	7	9	
	L	1	0	
	B/L	1	0	
Lap procedures (mean)		68	44.4	p = 0.49
Robotic procedures		0	0	

### Primary outcomes by task

#### Sea spikes

**Fig. 2:**
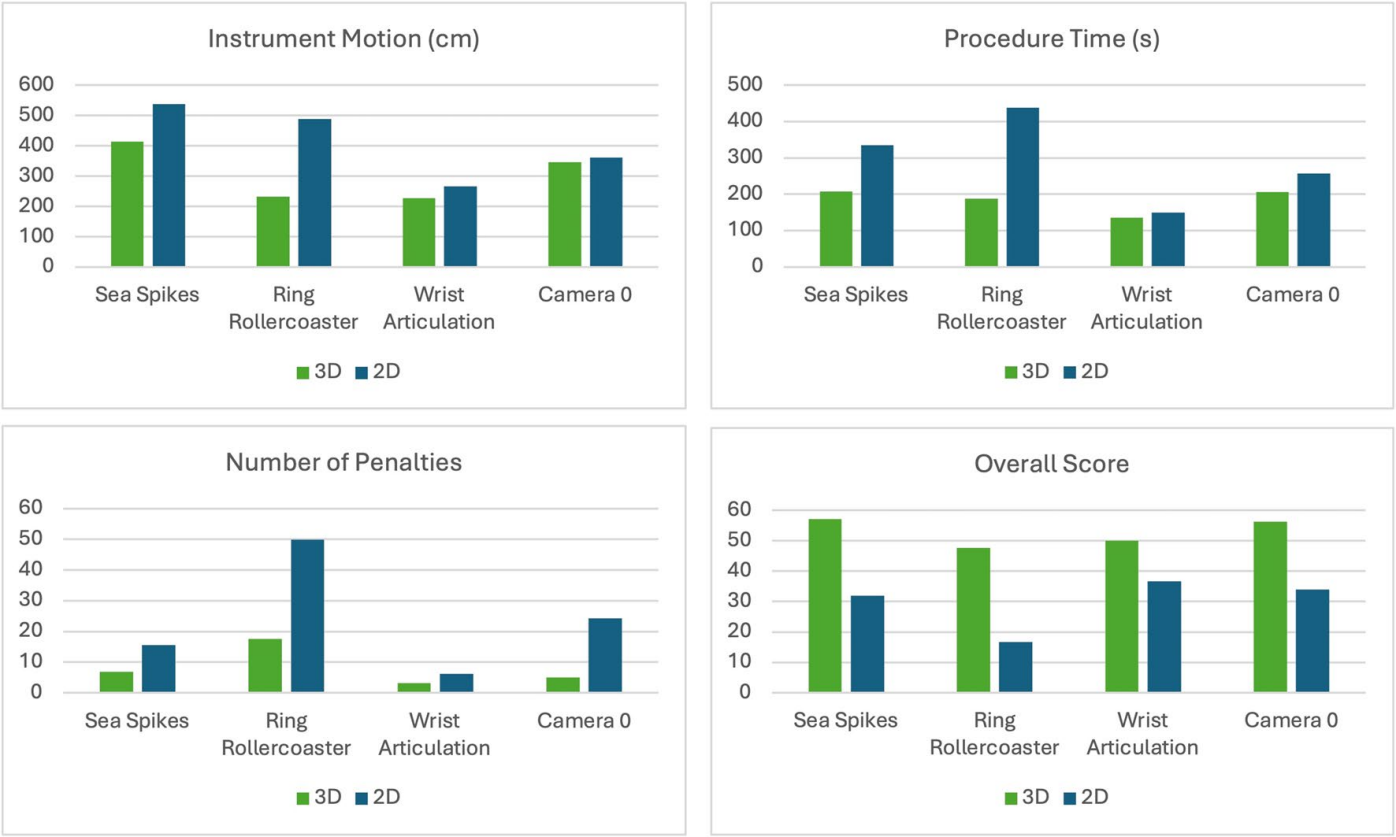
Mean differences per automated performance metric, by task

The Sea Spikes task revealed significant differences between the 3D VR and 2D video groups across the primary outcome measures (Fig. 2). The mean overall performance score for the 3D VR group was 56.1, compared to 31.9 for the 2D group (p = 0.02). The economy of motion was more efficient in the 3D VR group (mean = 414.5 cm) than in the 2D group (mean = 533.9 cm) (p = 0.04). The time to complete was significantly shorter for the 3D VR group (mean = 207.6 min) compared to the 2D group (mean = 334.3 min) (p = 0.01). Additionally, the penalty scores were lower in the 3D VR group (mean = −6.5) than in the 2D group (mean = −15.8) (p = 0.04).

#### Ring rollercoaster

For the Ring Rollercoaster task, the 3D VR group also demonstrated superior performance compared to the 2D video group. The mean overall performance score for the 3D VR group was 47.6, while the 2D group had a mean score of 16.7 (p = 0.04). The economy of motion was better in the 3D VR group (mean = 234.0 cm) compared to the 2D group (mean = 488.3 cm) (p = 0.04). The time to complete the task was shorter for the 3D VR group (mean = 189.4 min) compared to the 2D group (mean = 438.0 min) (p = 0.04). Although the difference in penalty scores was not statistically significant, the 3D VR group incurred fewer penalties (mean = −17.6) than the 2D group (mean = −49.8) (p = 0.23).

#### Wrist articulation

The wrist articulation task showed no statistically significant differences between the 3D VR and 2D video groups across the primary outcome measures. The mean overall performance score for the 3D VR group was 47.6, compared to 36.7 for the 2D group (p = 0.34). The economy of motion was slightly better in the 3D VR group (mean = 226.8 cm) than in the 2D group (mean = 266.5 cm) (p = 0.27). The time to complete the task was also shorter for the 3D VR group (mean = 148.1 min) compared to the 2D group (mean = 170.2 min) (p = 0.56). Lastly, the penalty scores were lower in the 3D VR group (mean = −4.6) than in the 2D group (mean = −6.9) (p = 0.16).

#### Camera

The analysis of the camera targeting task revealed mixed results between the 3D VR and 2D video groups across the primary outcome measures. The mean overall performance score for the 3D VR group was 56.3, compared to 34.8 for the 2D group (p = 0.14), indicating no statistically significant difference. The economy of motion was slightly better in the 3D VR group (mean = 336.3 cm) than in the 2D group (mean = 362.3 cm) (p = 0.69), also not statistically significant. The time to complete the task was shorter for the 3D VR group (mean = 208.6 min) compared to the 2D group (mean = 253.2 min) (p = 0.08), approaching significance. The penalty scores were significantly lower in the 3D VR group (mean = −5.9) than in the 2D group (mean = −24.3) (p = 0.04).

#### Overall

Across all tasks, the mean overall performance score for the 3D VR video group was 52.75, while the 2D video group had a mean score of 29.78 (p < 0.001) (Table 5). The mean instrument path length for the 3D VR video group was 305.09 cm, compared to 413.72 cm for the 2D video group (p < 0.01). The mean completion time for the 3D VR video group was 183.59 s, while the 2D video group took an average of 294.72 s (p < 0.01). The 3D VR video group incurred fewer penalty scores, reflecting fewer errors during the tasks. The mean penalty score for the 3D VR video group was −8.16, compared to −23.99 for the 2D video group (p = 0.03).

**Table 5:** Mean differences between 2 and 3D group

Category	3D mean	2D mean	P value
Overall	52.75	29.78	0.00090
Motion	305.09	413.72	0.00819
Time	183.59	294.72	0.00271
Penalty	-8.16	-23.99	0.03026

### Secondary outcomes

The NASA Task Load Index (NASA TLX) scores indicated no significant difference in perceived mental workload between the two groups. The mean NASA TLX score for the 3D VR video group was 45.3 (SD = 8.7), while the 2D video group had a mean score of 47.8 (SD = 9.2) (p = 0.42). In the 3D VR video group, 3 participants (21.4%) reported mild symptoms of cybersickness, such as slight dizziness and nausea. These symptoms were transient and resolved within a few minutes after removing the VR headset. No participants in the 2D video group reported any symptoms of cybersickness.

## Discussion

The results of this initial feasibility RCT indicate that 3D VR video acclimatisation significantly enhances simulator performance among novice robotic surgeons across initial introductory simulator tasks. Participants in the 3D VR group consistently demonstrated superior overall performance scores, economy of movement, faster completion times, and fewer errors compared to those in the 2D video group. Moreover, the NASA Task Load Index (NASA TLX) scores indicated no significant difference in perceived mental workload between the two groups, suggesting that the 3D VR video did not increase cognitive strain despite the enhanced performance.

One potential explanation for the enhanced performance observed in the 3D VR group is the activation of different brain areas associated with 3D visualization. Research has shown that 3D video can engage the parietal and occipital lobes more effectively than 2D video, leading to improved spatial awareness and depth perception. Studies utilizing electroencephalography (EEG) have demonstrated that brain activity exhibits higher global efficiency during 3D video viewing compared with 2D [9]. The impact of 3D video on anatomical brain lobe activation includes significant engagement of the prefrontal and occipital lobes, along with an increase in oxyhaemoglobin concentration in the prefrontal lobe [10]. These findings suggest enhanced neural processing and connectivity, indicating that 3D visualisation may facilitate more effective cognitive and perceptual integration. Furthermore, this likely contributes to the superior performance in tasks requiring spatial navigation, such as pick and place tasks.

Additionally, cognitive load theory provides a framework that may also explain the benefits of 3D VR acclimatisation. This theory delineates working memory into three distinct components: intrinsic load, extraneous load, and germane load [11]. Intrinsic load refers to the inherent complexity of the task to be learned. Extraneous load encompasses external factors that divert attention. Germane load refers to integration of new information with existing knowledge stored in long-term memory. Cognitive overload may occur when there is an excessive burden in any of these categories, thereby impeding the learning process. According to this theory, working memory has limited capacity, and excessive cognitive load can hinder learning and performance. The immersive nature of 3D VR video may reduce extraneous cognitive load by providing a more intuitive and engaging learning environment, allowing participants to focus more effectively on the task at hand. This reduction in cognitive load may have contributed to the improved efficiency and accuracy observed in the 3D VR group.

Furthermore, the trend towards better performance in the 3D VR group may be attributed to the increased realism and interactivity of the training environment. Prior exposure to a robotic procedure may allow individuals to understand the mechanics of wristed instruments better. This may be exaggerated through the use of 3D which gives the observer an improved appreciation of wrist manipulation. Consequent improvements to motor skills and hand–eye coordination may ensue. The positive feedback from participants regarding the immersive experience and perceived benefits of 3D visualisation supports this explanation.

## Limitations

Despite the promising results, it is important to acknowledge the limitations of this study. The sample size was relatively small, and the study was conducted in a controlled simulation environment. There is high risk of a type 2 error. However, the primary purpose of the trial was to assess feasibility with respect to study logistics and hardware considerations. Full learning curves were beyond the scope of this initial study and should be factored into subsequent trials. Technological and budget constraints meant that a previous generation VR headset was used. There have since been improvements in technology with newer headsets coming to market that may ameliorate problems such as cybersickness. Larger sample sizes and assessing impact upon the clinical setting with patient outcome data would needed to confirm these findings and explore the long-term impact of 3D VR video training.

## Conclusion

This study establishes the technical feasibility with respect to hardware and software constraints within an RCT setting. Perceived mental workload was comparable between the two groups, and the incidence of cybersickness was acceptable. Furthermore, this provides randomised data that 3D VR video acclimatisation can significantly enhance simulator performance amongst a small group of novice robotic surgeons. This was across all domains of automated performance metrics. The activation of different brain areas, reduction in cognitive load, and increased realism of the training environment could be factors contributing to the observed benefits. The foundations for a larger trial are laid, with these initial findings supporting the possible integration of 3D VR video prior to standard robotic surgery training protocols.
